# Plant-Based Diet and Pregnancy-Related Disorders: A Narrative Review

**DOI:** 10.1007/s13668-026-00759-z

**Published:** 2026-04-16

**Authors:** Ferhan Celik, İnci Türkoğlu, Koray Görkem Saçıntı, Ramazan Bülbül, Nazlı Tunca Sanlier, Nevin Sanlier

**Affiliations:** 1 Department of Nutrition and Dietetics, Gaziantep University School of Health Sciences, Gaziantep, 27310 Turkey; 2 Department of Nutrition and Dietetics, Hacettepe University School of Health Sciences, Ankara, 06100 Turkey; 3 Department of Obstetrics, Gynecology and Reproductive Sciences, Yale School of Medicine, New Haven, CT 06510 USA; 4 Division of Epidemiology, Department of Public Health, Faculty of Medicine, Hacettepe University, Ankara, 06100 Turkey; 5 Department of Obstetrics and Gynecology, Faculty of Medicine, Aksaray University, Aksaray, 68100 Turkey; 6 Department of Obstetrics and Gynecology, Turkish Ministry of Health, Niğde Çiftlik State Hospital, Niğde, 51800 Turkey; 7 Department of Nutrition and Dietetics, Ankara Medipol University School of Health Sciences, Ankara, 06050 Turkey

**Keywords:** Plant-based diet, Pregnancy, Gestational diabetes mellitus, Hypertension, Anemia, Nutrition

## Abstract

**Purpose of Review:**

Plant-based diets (PBDs), characterized by minimal or no intake of animal-derived foods and increased consumption of vegetables, fruits, whole grains, legumes, soy products, nuts, seeds, and plant-based oils, have garnered attention for their health benefits and environmental sustainability. While substantial evidence links PBDs to reduced risk of chronic diseases, their role in pregnancy-related disorders remains underexplored. This narrative review aims to evaluate the impact of PBDs on pregnancy-related disorders, including gestational diabetes mellitus (GDM), hypertensive disorders of pregnancy (HDP), anemia, and overall maternal and fetal outcomes.

**Recent Findings:**

Evidence suggests that adherence to PBDs—rich in whole grains, legumes, fruits, and vegetables—is associated with lower risks of GDM and HDP in several observational studies. While some studies report increased risk of low birth weight and anemia, others show no significant differences in maternal or fetal outcomes compared to omnivorous diets. Deficiencies in key nutrients—especially vitamin B12, iron, and omega-3 fatty acids—are a concern in poorly planned PBDs. Adequate supplementation and higher educational status support better pregnancy outcomes.

**Summary:**

Well-planned PBDs may reduce the risk of certain pregnancy complications and support maternal–fetal health when combined with adequate micronutrient intake. However, the evidence remains heterogeneous, and further high-quality longitudinal and interventional studies are warranted to establish the long-term safety and efficacy of PBDs in pregnancy.

## Introduction

A plant-based diet (PBD) is characterized by the exclusion or reduction of animal-derived foods and increased intake of vegetables, fruits, whole grains, legumes, soy products, nuts, seeds, and plant-based oils [1, 2]. A well-structured PBD typically provides higher amounts of dietary fiber, certain minerals, vitamins, and phytochemicals, while being lower in saturated fats and cholesterol compared to omnivorous diets [1, 2]. Evidence suggests that adherence to PBDs—including vegetarian and vegan dietary patterns— is connected to a diminished risk of several long-term health issues, including hypertension, cardiovascular diseases, type 2 diabetes, and particular cancers [3].

The growing popularity of PBDs in high-income countries has been driven not only by their recognized health benefits and potential to improve longevity but also by their environmental sustainability. PBDs have been linked to reduced greenhouse gas emissions, land use, and water consumption, aligning with global environmental conservation goals [2].

The global prevalence of vegetarianism varies significantly. In India, nearly 30% of the population follow vegetarian diets, mainly for cultural and religious reasons [3]. In contrast, vegetarianism is reported by approximately 10% of individuals in other parts of the world. In the United States, vegetarians and vegans account for about 5% and 2% of the population, respectively, with higher adherence among younger individuals [4, 5].

Although various professional organizations have endorsed PBDs, there is no universal consensus on their appropriateness for all life stages, particularly during pregnancy. The Academy of Nutrition and Dietetics (AND) has suggested that a vegan, lacto-vegetarian, and lacto-ovo-vegetarian diet, if well-planned, it is suitable for every phase of life, encompassing pregnancy and breastfeeding [1]. In the 2015–2020 Dietary Guidelines for Americans, the vegetarian diet was presented as one of three healthy eating patterns and customized meal plans were offered for both lacto-ovo-vegetarian and vegan lifestyles [1]. The 2019 EAT-Lancet Commission report has advocated a global shift to a PBD to optimize human health and promote environmental sustainability worldwide [6]. In contrast, the German Nutrition Society (DGE) has taken the position that it is difficult to provide sufficient nutrients with a vegetarian or vegan diet and has advised against recommending a vegan diet for pregnant women, breastfeeding mothers, infants, children or adolescents [7].

A key consideration is that emerging evidence distinguishes between healthful and unhealthful plant-based dietary patterns. Diets rich in whole plant foods—such as vegetables, legumes, whole grains, and nuts—are associated with favorable health outcomes. In contrast, PBDs high in refined carbohydrates and sugar-sweetened foods may pose nutritional risks, particularly during pregnancy. This distinction emphasizes the importance of assessing not only whether a diet is plant-based, but also the quality and composition of that diet [2]. Building on this, the health-promoting effects of PBDs are ultimately contingent upon adequate intake of key nutrients, including antioxidants, fiber, phytochemicals, and certain vitamins and minerals [2, 8].

Despite growing public interest and the promotion of PBDs as beneficial, their impact on pregnancy-related disorders remains an area of ongoing research. Given the unique physiological demands of pregnancy and the potential for both benefit and harm, it is essential to critically evaluate existing evidence. This narrative review aims to assess the impact of PBDs on pregnancy-related disorders, including gestational diabetes mellitus (GDM), hypertensive disorders of pregnancy (HDP), anemia, and overall maternal and fetal outcomes.

## Materials and Methods

This narrative review was conducted to synthesize current evidence on the association between plant-based diets (PBDs) and pregnancy-related outcomes. A comprehensive literature search was conducted using PubMed/MEDLINE, Cochrane Library, CINAHL, Scopus, and Web of Science, covering studies published up to December 12, 2024. The following keywords were used: “plant-based diet” AND (“gestational diabetes” OR “gestational hypertension” OR “pre-eclampsia” OR “anemia” OR “maternal outcomes” OR “fetal outcomes” OR “nutrient deficiencies”). Additionally, reference lists of included articles were manually screened for relevant studies.

Eligible studies met the following criteria: (1) original research articles, systematic reviews, meta-analyses, or letters to the editor; (2) focused on the association between plant-based diets (PBDs) and pregnancy-related outcomes; and (3) published in English with full-text availability. Studies published in other languages or deemed irrelevant based on title or abstract were excluded. Full texts of potentially relevant studies were assessed, and disagreements were resolved by consensus among the reviewers.

### Definition and Classification of Plant-Based Diets

In this narrative review, plant-based diets were broadly defined as dietary patterns that emphasize consumption of plant-derived foods—such as vegetables, fruits, legumes, whole grains, soy products, nuts, seeds, and plant-based oils—while reducing or excluding intake of animal-based foods. This definition was determined a priori based on widely accepted descriptions in the literature [9, 10]. To ensure consistency in study selection, a study was classified as evaluating a PBD if it met at least one of the following criteria:

- The diet was explicitly labeled as “plant-based,” “vegan,” “vegetarian,” or a closely related term by the authors.
- The dietary composition, as described in the methods or dietary assessment tools, reflected a predominance of plant-derived foods and minimal to no consumption of animal products.
- The study used a recognized plant-based dietary index (e.g., overall PBDI, healthful PBDI, unhealthful PBDI) as a primary exposure variable.

Studies in which the dietary exposure was ambiguous or plant-based eating was only a secondary or poorly defined component were excluded.

### Rationale for Methodological Approach

Given the heterogeneity in study designs, populations, and dietary definitions, a narrative review approach was selected rather than a formal systematic review, in order to accommodate the breadth and variation in the available literature. This methodology allowed for integration of findings from diverse study designs and dietary classifications while still maintaining clear parameters for what constituted a plant-based dietary pattern. Comparison groups generally included omnivorous, Western-style, or animal protein–centered diets.

## Mechanisms of Benefits and Risks of Nutrient Deficiencies in PBDs

The beneficial effects of PBDs on weight management, glycemic control, lipid profile, inflammation, and gut microbiota are mainly linked to their rich content of fiber, unsaturated fats, plant stanols and sterols, antioxidant vitamins, minerals, and phenolic compounds [1, 11]. For instance, fiber enhances satiety and reduces serum cholesterol and blood glucose levels [12, 13], while magnesium and chromium contribute to insulin function and glucose metabolism [12]. Plant sterols and stanols lower LDL-cholesterol by inhibiting its intestinal absorption [14]. Unsaturated fats and phenolics promote vascular health and reduce oxidative stress [15, 16]. Additionally, antioxidant vitamins and other bioactive compounds play a role in modulating inflammation, whereas fiber, prebiotics, and phenolics promote a favorable gut microbiota profile through the production of short-chain fatty acids (SCFAs) [17–19]. Table 1 outlines the nutrient-specific mechanisms underlying these health effects.

**Table 1:** Impact of PBDs on women’s health: bioactive compounds and mechanistic insights

Advantageous Outcomes	Component	Potential Mechanism
Weight Loss/The Maintenance of An Optimal Weight	Fiber	Dietary fiber adds bulk to the diet without the addition of digestible calories, resulting in higher satiety and weight loss [13]
Improved Glycemic Control	Fiber	Lowers glycemic index (Prevent blood-sugar spikes by slowing digestion and reducing the rate of glucose absorption into the bloodstream) [12]
	Phenolic bioactive components	Protection against oxidative stress, improving insulin sensitivity and pancreatic β cell activity, reducing hepatic glucose output [16]
	Magnesium, chromium	Act in glucose metabolism and insulin function [12]
Improved Lipid Profile and Vascular Health	Fiber	Dietary soluble fiber binds to bile acids in the small intestine, increasing excretion of the bile salts in the feces, thereby reducing blood lipids, cholesterol and blood glucose [13]
	Low Saturated Fat	Decrease total cholesterol and low density lipoprotein (LDL)- cholesterol levels [15]
	High Unsaturated Fat	
	Phenolic bioactive components	Protection against oxidative stress and improving vascular health [16]
	Plant stanols and sterols	Decrease low density lipoprotein (LDL)-cholesterol [14]
Reducing Inflammation	Vitamins C, E, beta carotene	Protection against oxidative stress [20]
	Phenolic bioactive components	
Modulation of the Gut Microbiota	Fiber	Maintain balanced the gut microbiota and has also an anti-inflammatory effect through the production of short-chain fatty acids (SCFAs) [17–19]
	Prebiotics	
	Phenolic Bioactive components	

Despite these advantages, nutritional concerns may arise if PBDs are not appropriately planned. A systematic review of 141 studies revealed that while PBD followers typically consume higher levels of fiber, folate, magnesium, and antioxidant vitamins, they may have lower intakes of vitamin B12, eicosapentaenoic acid (EPA), and docosahexaenoic acid (DHA) compared to omnivores [21]. Additional risk nutrients include iron, zinc, calcium, iodine, selenium, and vitamin D, especially during pregnancy—a period marked by increased nutrient demands for fetal development and maternal health [3, 22]. Several studies have raised concerns about potential deficiencies associated with poorly planned PBDs during pregnancy and early development [1, 7].

Several of these micronutrients are crucial for pregnancy outcomes. Vitamin B12, primarily found in animal-derived foods, is essential for fetal neural development, cognitive function, and growth; its deficiency may lead to complications such as spontaneous abortion, fetal growth restriction, and irreversible neurodevelopmental damage [23]. EPA and DHA, long-chain omega-3 fatty acids, are vital for neurodevelopment and immune regulation, yet their bioavailability is limited in plant-based diets due to poor conversion from ALA [24–26]. Iodine is crucial for thyroid hormone synthesis and fetal brain development, and even mild deficiency has been linked to lower cognitive performance in school-aged children [27–30]. Selenium and zinc contribute to antioxidant defense and immune function, and both inadequate and excessive levels may increase the risk of adverse pregnancy outcomes such as preeclampsia and preterm birth [31–36]. Vitamin D, essential for calcium homeostasis and placental health, is commonly low in vegetarian and vegan populations, increasing the risk of intrauterine growth restriction and other complications [37, 38]. Ensuring adequate intake of these nutrients through careful planning, supplementation, or fortified foods is particularly important for pregnant individuals following a plant-based diet. Table 2 lists plant-based sources of these nutrients and their physiological relevance.

**Table 2:** Dietary sources of critical nutrients in plant-based eating patterns

Nutrients	Physiological Roles	Plant-Based Sources
Protein	Growth, maintenance, and tissue development; involved in structural (e.g., collagen, keratin) and functional (e.g., enzymes, hormones) roles [39]	Tofu, lentils, peas, nuts, soybeans and soy products, lupins, quinoa, amaranth, buckwheat, horse beans, seitan, tempeh [40, 41]
Omega 3 fatty acids	Neurodevelopment, visual acuity, and cell membrane function [3]	Linseeds (or flaxseeds), soybean oil, rapeseed oil, tofu, walnuts, canola oil, hemp seed oil, pumpkin seeds, purslane and microalgae [22, 42], DHA enriched soy milk and breakfast bars [43], algae-based supplements [3]
Vitamin A	Cell division, fetal organ development, and immune function. Excess intake may cause fetal teratogenic effects [24]	Green leafy vegetables, yellow/orange vegetables or fruits [42]
Vitamin B2	Energy metabolism, antioxidant status, iron metabolism, and one-carbon metabolism [44]	Fortified breakfast cereal, fortified soya drink, almonds [42]
Vitamin B12	Hematopoiesis, DNA synthesis, homocysteine metabolism, myelin integrity and neurological development of the fetus [24, 45]	Fortified breakfast cereal, yeast extract, fortified soya drink [42]
Folic acid	Neural tube formation, DNA synthesis, homocysteine metabolism and prevention of birth defects [24]	Dark-green leafy vegetables, fruits such as oranges, mandarins, legume and nuts [42]
Vitamin D	Calcium and phosphate homeostasis, skeletal development, and immune support [24]	Fortified breakfast cereal, fortified soya drink, all margarines, other fortified fat spreads [42]
Calcium	Fetal bone and teeth mineralization, muscle function, and maternal bone health [46]	Fortified soya drink, sesame seeds, white/brown bread, fortified fruit juice, dried figs, broccoli, green leafy vegetables (except spinach), molasses, beans and pulses, tofu, soya mince [42]
Zinc	DNA and protein synthesis, enzymatic activity, cell division, immune, and wound healing [42]	Tofu, legumes (e.g. baked beans, chick peas, lentils), peas, nuts, seeds (e.g. cashew nuts, sunflower seeds) wholegrain cereals, and wholemeal bread [42]
Iron	Hemoglobin synthesis, oxygen transport, and prevention of maternal anemia [47]	Fortified breakfast cereal, wholemeal bread, dried fruit (e.g. apricots, prunes, raisins), green leafy vegetables, beans and pulses, molasses, nuts and seeds (almonds, pumpkin seeds, sesame seeds), tofu [42]
Selenium	Antioxidant defense, thyroid hormone metabolism, protection against oxidative stress, and reduction of pregnancy complications such as miscarriage, and preterm birth [34, 48]	Brazil nuts, sunflower seeds, molasses, wholemeal bread [42]
Iodine	Thyroid hormone production, thermogenesis, brain development, and metabolic regulation [47]	Iodized salt, seaweed [42]

To address these risks, national and international organizations offer dietary guidance. The Academy of Nutrition and Dietetics (AND) recommends diverse menu planning strategies that include legumes, tofu, nuts, seeds, and fortified foods to ensure nutritional adequacy in vegetarian diets [3, 43, 49]. Messina’s guide for North American vegetarians similarly emphasizes the inclusion of fortified cereals, plant-based dairy alternatives, and regular servings of legumes, whole grains, and dark green vegetables to meet the needs for iron, vitamin B12, calcium, and vitamin D [50]. Protein intake may also require adjustment, as plant proteins generally have lower bioavailability. Adult women are advised to consume approximately 1 g/kg/day of protein, a 20% increase over standard recommendations [3], and pregnant vegetarians may need up to 25 g additional protein daily to meet gestational requirements [3, 51]. AND further states that consuming a variety of plant-based proteins throughout the day ensures adequate amino acid intake without the need for combining complementary proteins within a single meal [43]. To simplify meal planning, Venti et al. proposed a modified food guide pyramid for vegetarians and vegans, prioritizing whole grains, fruits, vegetables, legumes, and fortified products, while recommending supplements for B12, D, and calcium at the top of the pyramid [3, 52].

Appropriate supplementation plays a central role in addressing potential nutrient gaps in PBDs, particularly during pregnancy, when micronutrient requirements are significantly elevated. Recent cross-sectional studies suggest that vegans can achieve adequate intakes of iron, vitamin B12 (through supplementation), and omega-3 fatty acids—particularly alpha-linolenic acid—at levels comparable to or even exceeding those of omnivores [53–55]. Moreover, one study found no adverse cognitive outcomes in children of vegetarian mothers despite their low intake of animal-derived EPA and DHA, potentially due to the protective effects of supplementation and overall healthier lifestyle patterns [56].

Global strategies also emphasize supplementation to prevent nutrient deficiencies. The World Health Organization (WHO) suggests that every pregnant woman, no matter her dietary habits, should take 30–60 mg of elemental iron along with 400 μg of folic acid on a daily basis to prevent conditions such as anemia, puerperal sepsis, preterm birth, and low birth weight [57]. When daily iron intake results in adverse effects, and in demographics where less than 20% of pregnant women are anemic, it is suggested that these women take 120 mg of elemental iron along with 2800 µg (2.8 mg) of folic acid weekly to improve outcomes for both mothers and newborns [57]. Expectant mothers with indications of vitamin D deficiency could be advised to take a daily vitamin D supplement of 200 IU (5 µg), especially in populations with limited sun exposure [58]. In regions where dietary calcium is insufficient, pregnant women are recommended to take daily calcium supplements (1.5–2.0 g of elemental calcium) to reduce the likelihood of developing pre-eclampsia [57]. Furthermore, vitamin A supplementation is specifically suggested for pregnant women in areas where vitamin A deficiency is a major public health challenge, aimed at preventing night blindness [57]. WHO advises against the routine use of vitamin B6, zinc, vitamin D, vitamin E, and multi-nutrient supplements for pregnant women [57].

Current evidence indicates that well-planned plant-based diets, supported by appropriate supplementation when necessary, are considered safe during pregnancy [3]. In addition to preventing potential nutrient deficiencies, guidance from nutrition and dietetics professionals—particularly on food sources, purchasing, preparation, and dietary adjustments—plays a key role in helping individuals following PBDs meet their nutritional needs [43].

## Impact of PBDs on Pregnancy-Related Disorders

### Gestational Diabetes Mellitus (GDM)

GDM, characterized by glucose intolerance that emerges or is first identified during pregnancy, impacts around 1–30% of pregnant individuals worldwide [59]. GDM is linked to a range of negative outcomes for both mothers and newborns, which encompass heightened incidences of infections, premature births, and cesarean sections, in addition to long-term complications such as type 2 diabetes and cardiovascular conditions. Fetus of mothers with GDM are predisposed to complications such as hypoglycemia, macrosomia, neonatal respiratory distress, congenital anomalies, fetal demise, and long-term metabolic disturbances [59, 60].

Epidemiological studies indicate that adjustments to one’s lifestyle, which encompass a balanced diet and routine physical activity, and preventing overweight, are crucial for managing GDM, and while PBDs may lower GDM risk, evidence supporting this is limited [1, 59]. A study focusing on a population cohort in China has analyzed the link between the Plant-Based Diet Index (PDI) and the risk of developing GDM. The study concluded that women in the top quartile of PDI had a 57% lower risk of GDM compared to those situated in the bottom quartile [59]. A prospective cohort study with 14,926 women indicated that greater adherence to a healthy PBD prior to pregnancy is associated with a reduced risk of GDM [11]. Another prospective cohort study comprising 13,110 participants in Nurse’s Health Study II demonstrated that for each additional 10 g per day of total dietary fiber intake, there was an observed decrease in GDM risk by 26%. Similarly, each 5-g increase in cereal or fruit fiber intake corresponded to a 23% or 26% reduction in risk, respectively [61].

A systematic review encompassing 44 observational studies between 2016 and 2022 found that consuming fruits, vegetables, legumes, and eggs was associated with a reduced risk of GDM, while the consumption of processed meat and following a low-carbohydrate diet was corelated with an increased risk [62]. The overconsumption of red meat has been considered as a risk factor for GDM due to the its heme- iron content and other components like advanced glycation end products and organic pollutants [63]. In a case–control study of 460 pregnant women, 200 with GDM and 260 controls, it was observed that fruit juices, sugar-sweetened foods, refined grains, and potatoes, considered unhealthy plant-based foods, are connected with a greater likelihood of developing GDM [64]. The results of this case–control study emphasize the importance of considering not only the plant-based origin but also the overall nutritional profile of foods in assessing the risk of GDM [64].

The potential mechanisms underlying the beneficial effects of PBDs are thought to include lower energy density and glycemic index, which may promote weight loss or help maintain an optimal weight and improve glycemic control. Additionally, the high content of antioxidants and anti-inflammatory compounds in plant foods may help reduce systemic inflammation, while the increased fiber intake may positively influence gut microbiota composition [11]. PBDs are also characterized by higher intakes of fiber, potassium, and magnesium, along with lower intakes of saturated fat, energy, glycemic load, and heme iron, providing non-heme sources of iron instead [65]. However, when PBDs are overly restrictive, poorly balanced, or not adequately supported with micronutrient supplementation, they may lead to deficiencies in key nutrients such as vitamin B12, iron, zinc, iodine, calcium, and protein—potentially exerting detrimental effects on fetal growth and development [66]. Moreover, in a cross-sectional study of 913 expectant mothers, it was reported that lower plasma vitamin B12 concentrations are related to a higher risk of GDM [67]. Taken together, PBDs may appear to have a potential role in reducing the risk of GDM; however, further comprehensive research is warranted. Table 3 summarizes studies investigating the effects of PBDs on pregnancy-related conditions such as GDM, HDP, anemia, and various maternal and fetal outcomes.

**Table 3:** Studies about the effects of plant-based diets (PBDs) on pregnancy-related disorders

Studies About the Effects of Plant-Based Diets (PBDs) on Pregnancy-Related Disorders				
Gestational Diabetes Mellitus (GDM)				
Study	Objective of the Study	Study Design/Population	Definition of PBD	Key Outcomes
Zhang et al., 2006 [61]	To examine the impact of dietary fiber consumptions and dietary glycemic load on the GDM risk	Prospective Cohort (*n* = 13,110)	Total fiber from cereals, fruits and vegetables	↑Fiber intake: ↓GDM risk (23–26%); ↑GI + ↓fiber: ↑GDM risk (2.15x)
Bao et al., 2014 [68]	To prospectively examine the association of 3 pre-pregnancy low-carbohydrate dietary patterns (LCDs) with risk of GDM	Prospective Cohort (*n* = 21,411)	Plant-based LCD (vegetable protein, vegetable fat and carb) vs. animal-based (animal protein, animal fat and carb) LCD	Plant protein: ↓GDM risk (30–51%); animal protein: ↑risk (~ 50%)
Zamani et al., 2019 [64]	To examine the association between plant-based dietary patterns and the risk of GDM	Case–control (*n* = 460) (200 cases, 260 controls)	Plant-based diet index (PBDI)	↑PDI: ↓GDM risk (OR = 0.47); unhealthy/healthy PBDI not significant
Wang et al., 2020 [59]	To evaluate the association between overall plant-based diet index (PDI) and GDM risk, during the 13–28 weeks of pregnancy	Prospective Cohort (*n* = 2,099)	Plant-based diet index (PBDI)	Highest PDI quartile: 57% ↓GDM odds (OR = 0.43)
Kesary et al., 2020 [63]	To explore the association of vegetarian-vegan diets and pregnancy outcomes	Retrospective (*n* = 1419)	Vegan and vegetarian diets	Trend toward ↓GDM in vegans (aOR = 0.54); not significant in vegetarians
Chen et al., 2021 [11]	To investigate the relationship between pre-pregnancy adherence to a PBD and developing risk of GDM in a large population	Prospective Cohort (*n* = 14,926)	Plant-based diet index (PBDI)	↑healthy PBDI: ↓GDM risk (RR = 0.89); unhealthy PBDI not associated
Lambert et al., 2023 [62]	To synthesize the available evidence regarding the connection between GDM and maternal dietary components	Systemic Review	Fruits, vegetables, legumes, antioxidant-rich foods	↑Antioxidants and plant foods: ↓GDM risk
Zhu et al., 2023 [69]	To assess the impact of plant-based dietary patterns on the likelihood of developing GDM	Systematic Review & Meta-analysis (*n* = 32,006)	Vegetarian and vegan diets	↑PBD adherence: ↓GDM risk (RR = 0.88)
Przybysz et al., 2023 [70]	To compare maternal and neonatal outcomes between women adhering to a PBD and those following an omnivorous diet	Cross-Sectional (*n* = 1015)	Vegetarian and vegan diets	No impact of PBD on GDM; physical activity ↓GDM in omnivores only
Gao et al., 2023 [60]	To evaluate the effect of diet quality on the risk of GDM	Systematic Review and Meta-analysis (*n* = 108,084)	Vegetarian and vegan diets	↑PBD/healthy diets: ↓GDM risk; inflammatory/low-carb diets: ↑risk
Hypertensive Disorders of Pregnancy (HDP)				
Cartel et al., 1997 [71]	To investigate preeclampsia symptoms in the maternity care records of vegan mother	Retrospective Observational (*n* = 775)	Vegan diet	Very low preeclampsia rate (0.13%) among vegans
Frederick et al., 2005 [72]	To examine how maternal intake of dietary fiber, potassium, magnesium, and calcium relates to the risk of preeclampsia	Case–Control (*n* = 511)	High intake of fiber, K, Mg, Ca	↑Fiber & K intake: ↓PE risk (OR ~ 0.46–0.49)
Longo-Mbenza et al., 2008 [73]	To evaluate whether the incidence of pregnancy-induced hypertension is low and to determine if vegetable intake and physical activity offer protective effects against the onset of pregnancy-induced hypertension among rural women in the Democratic Republic of Congo	Longitudinal (*n* = 238)	Vegetable-rich diet & physical activity	Vegetarians: ↓PE (3.1% vs. 9.7%); ↑veg intake & activity protective
Qui et al., 2008 [74]	To evaluate the relationship between dietary fiber intake during early pregnancy and the risk of developing preeclampsia later in pregnancy in Washington State	Prospective (*n* = 1,538)	High fiber intake in early pregnancy	↑Fiber: ↓PE risk (RR = 0.28)
Piccoli et al., 2015 [75]	To examine existing literature on the effects of vegan and vegetarian diets on pregnancy outcomes	Narrative Review	Vegetarian and vegan diets	Results on HDP inconsistent across studies
Ikem et al., 2019 [76]	To investigation of the relationship between nutritional behaviour in mid-pregnancy and pregnancy-associated hypertension (PAH)	Prospective Longitudinal (*n* = 55,139)	Seafood diet (high consumption of fish and vegetables) vs. Western dietary patterns (high consumption of potatoes-including French fries, mixed meat, margarine and white bread)	Seafood: ↓GH & PE (OR ~ 0.79–0.86); Western diet: ↑GH & PE (OR ~ 1.18–1.40)
Mi et al., 2019 [77]	To examine the associations between dietary patterns during pregnancy and the risk of preeclampsia	Randomized Controlled (*n* = 987)	Vegetable-based dietary pattern (high consumption of mushrooms, leafy and cruciferous vegetables, root vegetables, melon vegetables and legumes)	↑Vegetable pattern: ↓PE & proteinuria risk
Raghavan et al., 2022 [78]	To analyze dietary patterns before and during pregnancy and risk of HDP	Systematic Review	Vegetables, legumes, fish vs. red meat, refined grains	↑Plant-based foods: ↓HDP (30–42%) & PE (14–29%)
Hedegaart et al., 2024 [79]	To investigate the correlations between various types of PBDs during pregnancy and their impact on birth outcomes and pregnancy complications	Prospective Observational (*n* = 91,381)	Vegetarian, vegan, omnivorous diets	Vegans: ↑PE incidence; ↓birth weight (− 240 g vs. omnivores)
Mitsunami et al., 2024 [80]	To assess the prospective relationship between adherence to PBDs prior to pregnancy and the risk of developing HDP	Prospective Cohort (*n* = 11,459)	PBD index prior to pregnancy	↑PBD index: ↓HDP risk (RR = 0.76); BMI mediated 39–48% of effect
Anemia				
Koebnick et al., 2001 [81]	To evaluate and contrast folate intake and status during pregnancy between women with a consistently high intake of vegetables and those following a typical Western diet	Longitudinal Case–Control (*n* = 109)	Ovo-lacto vegetarian vs. Western diet	↑Folate status in vegetarians; ↓risk of folate deficiency (OR = 0.10–0.52)
Sharma et al., 2003 [82]	To view the effect of various dietary habits on the prevalence of anemia in pregnant women	Cross-Sectional (*n* = 1,150)	Vegetarian vs. meat-based diets	Anemia prevalence high across all diets (~ 95–96%); no significant difference
Koebnick et al., 2004 [83]	To compare serum levels of vitamin B12 and homocysteine in pregnant women following a lacto-ovo-vegetarian diet, a low meat diet (< 300 g/week), or a high meat diet (> 300 g/week)	Longitudinal Cohort (*n* = 109)	Vegetarian vs. low/high meat intake	↓B12 in vegetarians; ↑homocysteine; risk of deficiency present
Alwan et al., 2011 [84]	To investigate the relationship between iron intake during pregnancy and birth size in the UK	Prospective Cohort Study. *n* = 1,274	Vegetarian vs. non-vegetarian	Vegetarians: ↓risk of low iron intake (OR = 0.5); ↑supplement use; iron intake ↑birthweight
Piccoli et al., 2015 [75]	To examine existing literature on the effects of vegan and vegetarian diets on pregnancy outcomes	Narrative Review	Vegan/vegetarian vs. omnivorous diets	Some ↑risk of B12/iron deficiency in vegans/vegetarians
Tan et al., 2019 [85]	To examine the association between a vegetarian diet during pregnancy and various maternal and fetal outcomes	Systemic review & meta-analysis	Vegetarian diets during pregnancy	Mixed evidence: some studies show ↑anemia risk, others no difference
Avnon et al., 2020 [86]	To assess the impact of maternal diets on the levels of vitamin B12, folic acid, ferritin, and hemoglobin in both maternal and umbilical cord blood	Prospective Observational (*n* = 273)	Vegan, vegetarian, omnivore	Vegans: ↓ferritin vs. pescatarians; supplements ↑cord B12 levels
Yisahak et al., 2021 [87]	To examine associations of vegetarianism during pregnancy with maternal and neonatal outcomes	Prospective Cohort (*n* = 1,948)	Vegetarian diet	↑SGA risk (OR = 2.51); ↑inadequate weight gain (OR = 2.24); anemia not directly assessed
Maternal and Fetal Outcomes				
Fikree et al., 1994 [88]	To investigate the prevalence and contributing risk factors of adverse pregnancy outcomes	Prospective (*n* = 755)	Vegetarian vs. omnivorous	Vegetarian mothers: ↑IUGR risk (RR = 2.3)
North et al., 2000 [89]	To investigate the impact of a vegetarian diet and phytoestrogen intake during pregnancy on the incidence of hypospadias	Prospective (*n* = 7,928)	Vegetarian diet	↑Hypospadias risk (OR = 4.99)
Philips F., 2000 [90]	To examine the outcomes associated with vegetarian nutrition during pregnancy	Briefing Paper	Vegetarian diet	↓Birthweight possibly due to low Fe, folate, B12
Akre et al., 2008 [91]	To explore the association between hypospadias and exposure to exogenous hormones, as well as maternal dietary patterns during pregnancy	Case–Control Prospective (*n* = 719)	No meat/fish diet	↑Hypospadias risk (OR = 4.6)
Ramon et al., 2009 [92]	To investigate the association between fruit and vegetable consumption during pregnancy and anthropometric measures at birth within a general population mother-infant cohort in Spain	Case–Control (*n* = 787)	Fruit & vegetable intake	↓Fruit/vegetable in 1 st trimester: ↑SGA risk (OR = 3.7)
Kort et al., 2011 [93]	To evaluate the impact of maternal dietary patterns on the incidence of hypospadias	Case–Control Prospective (*n* = 961)	Whole-food vs. poor-quality diet	↓Plant foods: ↑Hypospadias (OR = 1.54)
Fikawati et al., 2013 [94]	To assess the nutrient intake of vegetarian pregnant women in Indonesia and its relationship to pregnancy outcomes	Prospective (*n* = 85)	Vegetarian diet	↓Micronutrient intake; B12 linked to ↑birthweight
Larsen et al., 2014 [95]	To investigate the relationship between maternal vegetarianism and the likelihood of neurodevelopmental impairments in the Danish National Birth Cohort	Cohort (*n* = 80,743)	Vegetarian/vegan diet	Insufficient data on neurodevelopment outcomes
Murphy et al., 2014 [96]	To evaluate the relationships between fruit and vegetable consumption during pregnancy and both infant birth weight and small-for-gestational-age status	Systematic Review	Fruit & vegetable intake	↓Vegetable intake: ↑SGA risk; ↑fruit: ↑birthweight
Grieger et al., 2014 [97]	To identify associations between maternal dietary patterns during the 12 months preceding conception and their effects on fetal growth and the risk of preterm delivery in Australia	Retrospective (*n* = 309)	High protein/fruit vs. high fat/sugar diet	Protein/fruit: ↓preterm risk (OR = 0.31); high-fat: ↑preterm
Samtani et al., 2014 [98]	To assess the frequency of hypospadias in the Northern Indian region and to identify the associated risk factors	Case–Control (*n* = 211)	Vegetarian vs. omnivorous	Vegetarians: ↑Hypospadias risk (OR = 2.47)
Englund-Ögge et al., 2014 [99]	To investigate the potential link between maternal dietary patterns and the risk of preterm delivery	Prospective Cohort (*n* = 95,200)	Prudent diet (high intakes of vegetables, fruits, whole grains, and fiber-rich bread) vs. Western diet	↑Prudent/traditional diet: ↓preterm risk; Western: no effect
Rasmussen et al., 2014 [100]	To assess and define dietary patterns in the Danish National Birth Cohort through novel methods utilizing Principal Component Analysis	Prospective, Longitudinal Cohort (*n* = 60,000)	Western diet	↑Western diet score: ↑preterm birth (OR = 1.30)
Shrestha et al., 2016 [101]	To determine socio-economic and maternal reproductive factors affecting low birth weight (LBW) babies in Nepal	Cross-sectional and Observational (*n* = 350)	Vegetarian vs. omnivorous	↑LBW in vegetarians (52.8%) vs. omnivores (30.9%) (NS)
Agnoli et al., 2017 [102]	Evaluating the Viability of Vegetarian Diets During Pregnancy in Italy	Review	Vegetarian diet	↓DHA in infants born to vegetarian mothers
Crozier et al., 2019 [56]	To examine whether vegetarianism during pregnancy is linked to changes in maternal nutritional status and to cognitive function in children at the age of six to seven years	Prospective Observational (*n* = 3158)	Vegetarian diet	No link to ↓neurocognition; some ↓nutrient status
Kesary et al., 2020 [63]	To explore the association of vegetarian-vegan diets and pregnancy outcomes	Retrospective (*n* = 1419)	Vegan vs. omnivore	Vegan: ↓birthweight centile; ↑SGA risk (aOR = 1.74); BMI-adjusted NS
Yisahak et al., 2021 [87]	To examine associations of vegetarianism during pregnancy with maternal and neonatal outcomes	Prospective Cohort (*n* = 1,948)	Vegetarian diet	↑SGA (OR = 2.51); ↑inadequate weight gain (OR = 2.24)
Avnon et al., 2021 [103]	To assess the impact of a maternal vegan diet on pregnancy outcomes	Prospective Observational (*n* = 273)	Vegan vs. vegetarian/omnivore	Vegan: ↑SGA (RR = 5.9); ↓birthweight & weight gain

### Hypertensive Disorders of Pregnancy (HDP): Gestational Hypertension and Pre-eclampsia

HDP, including gestational hypertension, preeclampsia, and eclampsia, represent significant contributors to both maternal and perinatal morbidity and mortality. HDP is characterized by persistently elevated systolic (≥ 140 mmHg) and/or diastolic blood pressure (≥ 90 mmHg), verified through two separate readings obtained at a minimum interval of four hours following the 20th week of gestation [104]. Gestational hypertension refers to de novo hypertension without proteinuria, whereas preeclampsia is characterized by the presence of proteinuria or end-organ dysfunction. When seizures occur in the context of preeclampsia, the condition is classified as eclampsia [104].

Globally, HDP affects 5–10% of pregnancies and contributes to an estimated 76,000 maternal deaths and over 500,000 fetal and neonatal complications annually [104, 105]. In high-income countries, HDP accounts for approximately 16% of maternal deaths, according to the WHO [106]. Preeclampsia, in particular, can lead to multi-organ dysfunction, including cardiovascular, renal, hepatic, neurological, hematologic, and placental complications [107]. Established risk factors for HDP include advanced maternal age (≥ 35 years), pre-pregnancy overweight and obesity, excessive gestational weight gain, multifetal gestation, diabetes, alcohol consumption, and prior HDP [104]. A meta-analysis demonstrated that women with a pre-pregnancy body mass index (BMI) equal to or greater than 25 kg/m^2^ are approximately 3.9 times more likely to develop preeclampsia compared to those whose BMI is below 25 kg/m^2^ [104].

Obesity and preeclampsia share overlapping pathophysiological mechanisms, including insulin resistance, impaired trophoblast invasion, and defective spiral artery remodeling, which contribute to placental hypoxia and ischemia. This hypoxic environment triggers the production of mediators that are both antiangiogenic and pro-inflammatory in nature, leading to endothelial dysfunction and oxidative stress—central features of preeclampsia pathogenesis [108].

PBDs, characterized by high intakes of fiber, unsaturated fats, and phytochemicals, and low levels of saturated fats and added sugars, have demonstrated efficacy in improving metabolic health, including weight control and insulin sensitivity—two critical factors in the prevention of HDP [2, 109]. Moreover, a PBD low in arachidonic acid (AA) may confer additional protection by reducing thromboxane A2 production and supporting prostacyclin synthesis, which may be disrupted in preeclampsia [3].

Emerging evidence supports a protective association between PBDs and HDP. A 19-year longitudinal study involving 11,459 women reported a 24% lower risk of preeclampsia among individuals with high adherence to a plant-based dietary pattern, largely attributed to improved weight management [80]. Research from a Danish cohort comprising 55,139 pregnant women during the years 1996 to 2002 indicated that a Western dietary pattern, which includes potatoes, meat, bread, and solid fats, raises the likelihood of developing preeclampsia by 40% and gestational hypertension by 18% [76]. Conversely, a diet focused on seafood, featuring fish and vegetables, correlates with a 21% lower risk of preeclampsia and a 14% lower risk of gestational hypertension [76].

A systematic review that focused on various dietary approaches in pre-pregnancy and gestation reported that consumption of vegetables, fruits, nuts, legumes, vegetable oils, and fish instead of red/processed meat and refined grains is related to a 30–42% lower risk of hypertension disorders of pregnancy and a 14–29% lower risk of preeclampsia [78]. Additionally, consumption of diets rich in of plant-derived foods, through a daily intake of a minimum of three portions of vegetables, along with engaging in physical activity, appear to lower the risk of pregnancy-induced hypertension [71, 73]. A longitudinal study conducted in Congo showed that preeclampsia was more common in women with rare daily servings of vegetables (33.3%) than pregnant women with 3 or more daily servings of vegetables (3.7%) [73].

A case–control study involving 172 women diagnosed with preeclampsia and 339 healthy counterparts indicated that a dietary pattern emphasizing fruits, vegetables, whole grains, dark bread, and low-fat dairy products may lower the risk of developing preeclampsia, potentially attributable to reduced intake of fats and sugars and increased consumption of potassium and dietary fiber [72]. Plant-based dietary approaches typically furnish reduced levels of fat and sugar, along with increased fiber content [72]. Furthermore, PBDs may potentially reduce the risk of proteinuria, thereby being correlated with a lower incidence of pre-eclampsia. [77]. Despite some studies noting lower birth weights and potential vitamin B-12 and iron deficiencies in vegetarian and vegan mothers, others report higher birth weights; overall, while increasing maternal intake of plant-derived foods shows promise, additional studies are required to assess the impact of PBDs on preeclampsia [75].

### Anemia in Pregnancy

WHO defines pregnancy-related anemia as a hemoglobin level under 11 g/dL or a hematocrit level that does not exceed 33% at any gestational stage [110]. Anemia affects 52% of pregnant women in underdeveloped or developing countries compared to 20% in industrialized nations, with iron deficiency as the primary cause and folate and vitamin B12 deficiencies also common, especially in socioeconomically deprived areas [111]. During pregnancy, the physiological demand for iron increases substantially—approximately 1 g is required to support maternal and fetal erythropoiesis, placental growth, and compensatory blood volume expansion [112]. Inadequate iron status during gestation has been associated with unfavorable results such as intrauterine growth restriction (IUGR), low birth weight, and long-term neurodevelopmental impairments in offspring [111, 113]. Maternal risks include heightened susceptibility to infections, hemorrhage-related mortality, HDP, sepsis, and—in cases of severe anemia (hemoglobin < 5.0 g/dL)—cardiac failure [111, 113].

Concerns regarding iron status in individuals adhering to PBDs primarily stem from the absence of heme iron, the more bioavailable form of dietary iron, and the presence of absorption inhibitors such as phytic acid, tannins, chlorogenic acid, and certain soy proteins [114]. However, culinary techniques—such as sprouting, fermenting, preparing sourdough, soaking, and discarding soaking water—can reduce phytic acid content and improve non-heme iron bioavailability [115]. Concurrent intake of vitamin C-rich foods has also been shown to enhance iron absorption and is strongly recommended for vegetarians and vegans during pregnancy [115].

The EPIC-Oxford cohort study, recognized as one of the largest investigations focusing on vegetarian diets, comprised 31,546 non-pregnant individuals following vegetarian/vegan diets. The study revealed that vegans had the highest intake of iron and folate, but the lowest intake of vitamin B-12 [116]. A prospective cohort study carried out in the USA from 2009 to 2013, involving 1,948 pregnant women, revealed that vegetarians do not show a heightened risk of gestational anemia [87]. In a research study featuring 1,274 pregnant women from the United Kingdom, it was reported that vegetarians, who tend to be older, have a higher socioeconomic status, and take supplements, exceed an average daily iron intake of 14.8 mg. Additionally, vegans were less likely to have an iron intake of less than 14.8 mg per day and showed a higher propensity to use iron supplements during the first and second trimesters [84]. Moreover, emerging evidence suggests that vegans may exhibit enhanced non-heme iron absorption compared to omnivores, potentially due to physiological adaptations such as lower hepcidin levels [117].

A retrospective study of 85 vegetarian mothers found that their mean iron intake (9.3 mg/day) during pregnancy was below recommended levels and correlated with lower infant birth weight. [94]. A cross sectional study focused on risk factors for maternal anemia suggested that higher anemia prevalence is associated to vegetarianism, low income and low educational status [113]. According to the results of a study conducted in Delhi, the prevalence of anemia was found to be approximately 96% among both vegetarian and non-vegetarian pregnant women, with no significant difference in hemoglobin levels (mean 9.2 mg/dL) across the two dietary groups [118]. A thorough examination and meta-analysis of observational studies demonstrated that the risk of maternal anemia for vegetarian mothers remains uncertain due to varying results in the included studies, which showed high heterogeneity [85]. Adequate and appropriate nutritional supplementation is essential for all pregnant women [3]. The researchers have emphasized that elevated anemia rates were linked not only to nutritional factors but also to social determinants such as education, socioeconomic status, and government fortification strategies concerning food [119]. A recent large-scale cohort study from Japan investigated the association between plant-based diet indices (PBDIs) and maternal iron status in the third trimester of pregnancy. The findings revealed that higher adherence to an unhealthful plant-based diet index (uPDI)—characterized by increased intake of refined grains, sugary beverages, and other low-nutrient plant foods—was associated with an elevated risk of iron deficiency anemia. In contrast, no significant association was observed between a healthful plant-based diet index (hPDI) and anemia or iron deficiency. These results underscore the importance of diet quality in plant-based eating patterns during pregnancy, particularly regarding iron-related outcomes [120].

While restrictive or poorly planned PBDs—particularly in resource-limited settings—may increase the risk of iron-deficiency anemia, current evidence from high-income countries does not consistently support the hypothesis that adequately balanced and supplemented plant-based or vegetarian diets pose a significant risk for maternal anemia [115]. Thus, individualized nutritional counseling and routine monitoring, along with appropriate supplementation, remain critical components of prenatal care.

### Maternal and Fetal Outcomes

The impact of maternal adherence to plant-based or vegetarian diets on fetal development and perinatal outcomes remains a subject of ongoing investigation and debate. While certain observational studies have suggested associations between vegetarian diets during pregnancy and increased risk of low birth weight [101] and congenital anomalies such as hypospadias [89, 91, 98], others have proposed potential protective effects against intrauterine growth restriction [88]. Piccoli et al. reported that appropriately planned vegetarian diets can be safe for fetal development during pregnancy [75], and numerous studies find no substantial association between a vegetarian diet during this period and adverse outcomes [121–125].

A prospective cohort study conducted in the United States reported that while a vegetarian diet during gestation is linked to a reduced neonatal size, possibly due to the mothers’ decreased weight gain during pregnancy, there is no correlation with morbidities associated with being small for gestational age (SGA) [87]. The same research indicated that there were no notable correlations between vegetarian diets and the occurrence of GDM, HDP, or anemia. [87]. In a comprehensive prospective study conducted in Norway between 1999 and 2008, involving 114,500 children and 95,200 mothers, researchers examined the association between maternal dietary patterns and the risk of preterm birth. Three distinct dietary patterns were identified: Prudent (characterized by high intakes of vegetables, fruits, whole grains, and fiber-rich bread), Western (high in salty and sweet snacks, white bread, desserts, and processed meat products), and Traditional (marked by higher consumption of potatoes and fish) [99]. The Prudent diet was associated with a reduced risk of preterm, late, and spontaneous preterm deliveries, whereas no significant associations were observed for either the Western or the Traditional diet [99]. In a prospective observational study that included 273 women, the findings showed that infants born to vegan mothers had lower gestational weight gain, but within the normal range, compared to omnivores. Additionally, there is no difference between the groups regarding HDP and GDM [103]. A meta-analysis of 38 observational studies aimed to explore vegetarian diet during pregnancy and neonatal-maternal outcomes has emerged that an elevated risk among Asian mothers adhering to a vegetarian diet for delivering infants with low birth weight in comparison to their omnivorous counterparts [85].

Conversely, several prospective studies have examined the correlation between a Western diet, which is marked by elevated intake of meat products, added sugars, and saturated fats, along with reduced consumption of fruits and vegetables, and an increased incidence of low birth weight, preterm births, and SGA infants [100, 126–128]. However, it is important to acknowledge that weak associations may have been disregarded due to limited statistical power arising from small sample sizes [85].

A systematic review, which finally included 40 studies aimed at exploring the effects of maternal diets on birth outcomes, has reported that high consumption of vegetables, fruits, whole grains, and dairy products is associated with a reduced risk of preterm birth and SGA [129]. A prospective observational found that small-for-gestational-age incidence is significantly higher in vegans compared to omnivores, lacto-ovo-vegetarians, and pescatarians [103]. In Poland, a cross-sectional study including 1,015 pregnant women identified no link between a PBD and maternal or fetal outcomes like birth weight or GDM, and noted that an omnivore diet offers no greater advantages [70].

**Fig. 1:**
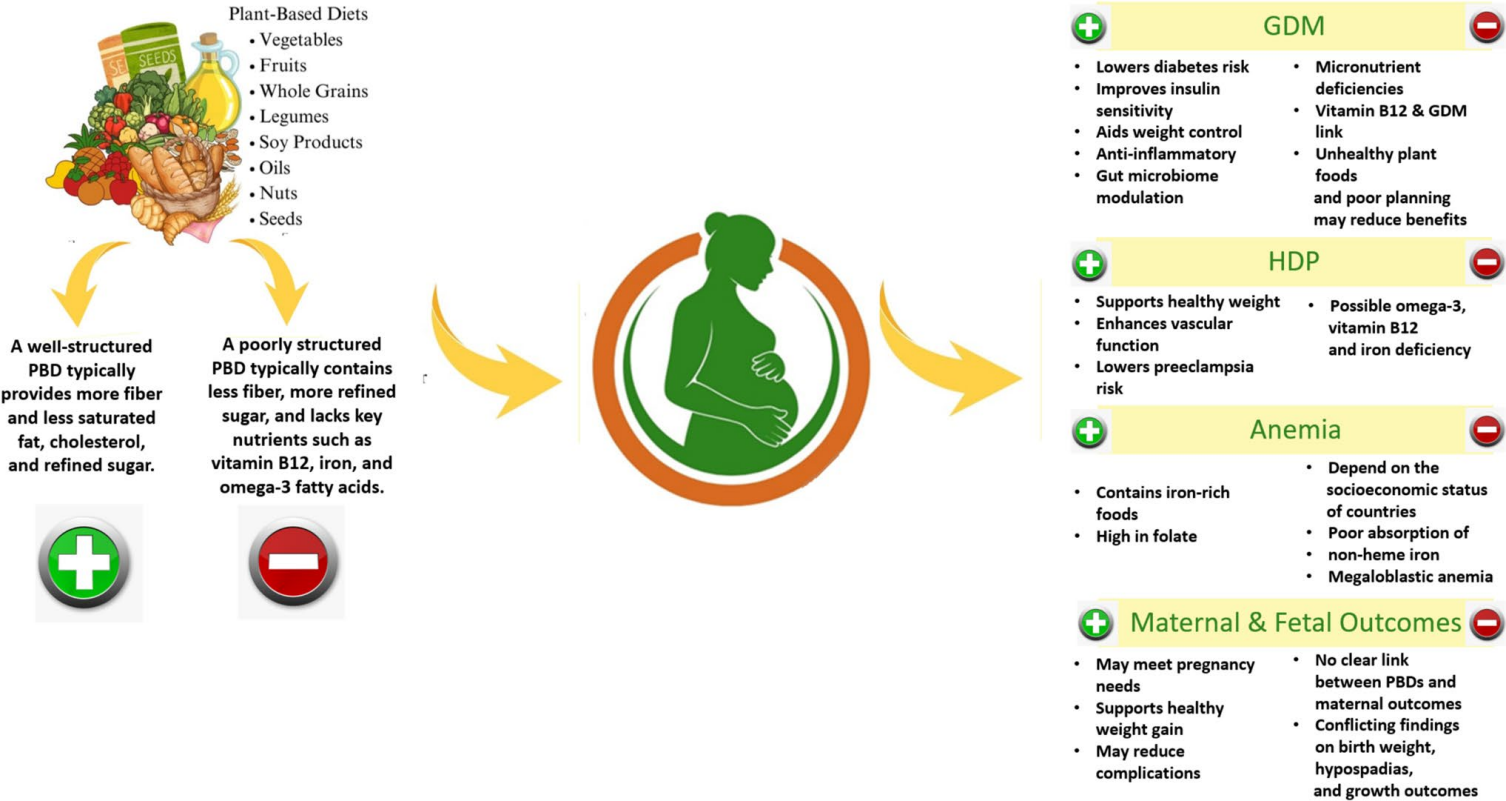
Potential advantages, nutritional risks, and pregnancy-related outcomes linked to PBD patterns in pregnancy

The theory of early life programming suggests that maternal nutrition can influence offspring health and chronic disease risk later in life [130]. A long-term prospective observational study aimed to investigate maternal diet during both in early and late pregnancy periods and its correlation with cognitive performance of their children at the ages of 6–7 [56]. The study has demonstrated that vegetarian pregnant women, compared with omnivore pregnant women, tend to take nutritional supplements in their pregnancy. Moreover, after delivery, the average duration of breastfeeding was 13–14 weeks longer in vegetarian mothers than omnivore mothers. Children who were born to women who adhered to a vegetarian diet during pregnancy had higher IQ scores compared to those born to omnivorous mothers [56]. These findings suggest that adherence to a well-planned PBDs during pregnancy may not impair, and may even support, neurodevelopmental outcomes. Figure 1 illustrates the potential advantages, nutritional risks, and pregnancy-related outcomes linked to PBD patterns when followed during pregnancy.

## Functional Plant-Based Nutrients and Research Directions

Emerging research into plant-derived sources of vitamin B12 highlights promising alternatives for individuals adhering to strict PBDs. Nori (Porphyra spp.), a commonly consumed seaweed in East Asian cuisine, has been identified as one of the richest plant-based sources of naturally occurring vitamin B12, along with providing omega-3 fatty acids and iron [131]. Approximately 4 g of dried nori—containing 77.6 μg of vitamin B12 per 100 g—can provide the recommended dietary allowance (RDA) of 2.4 μg/day [131]. However, vitamin B12 content in commercial nori products is highly variable, and not all products contain active forms of the vitamin; therefore, label checking is essential.

Shiitake mushrooms, another plant-based source, have also demonstrated variable but significant B12 content (1.3–12.7 μg per 100 g dry weight), in addition to ergocalciferol (vitamin D₂: 18.9 mg/100 g) and iron (2.0 mg/100 g) [131]. While functional foods such as nori and shiitake mushrooms may offer a potential means to improve micronutrient intake, their safety, bioavailability, and physiological effects—particularly during pregnancy—remain insufficiently studied and require further clinical investigation.

To ensure maternal and fetal health, adequate vitamin B12 intake is essential during pregnancy. The Institute of Medicine (IoM) recommends a daily intake of 2.6 µg, while the European Food Safety Authority (EFSA) suggests a higher intake of 4.5 µg for pregnant women [132, 133]. These recommendations may vary based on individual factors such as age, physiological status, and overall health [133].

Cyanocobalamin and methylcobalamin are the two most commonly used forms of vitamin B12. Methylcobalamin is the biologically active form, whereas cyanocobalamin requires conversion in the body before it can be utilized [134]. Pregnant women adhering to PBDs, particularly vegan diets, should receive clear guidance on effective supplementation strategies to prevent deficiency and support optimal health outcomes for both mother and fetus [133, 134].

## Limitations

This assessment offers a summary of the possible connections between PBDs and pregnancy-related disorders; however, several limitations must be acknowledged. First, the available body of literature on the direct impact of PBDs on specific pregnancy outcomes remains limited, restricting the ability to draw definitive conclusions. Importantly, the existing evidence is predominantly derived from observational studies including cohort, case–control, and cross-sectional studies; therefore, the findings should be interpreted as associations rather than causal effects. While these studies provide valuable insights into potential associations, they are inherently susceptible to residual confounding, selection bias, and reverse causality.

Further limitations include substantial heterogeneity in study designs, population characteristics, dietary assessment tools, and inconsistent definitions of PBDs (e.g., vegan, vegetarian, flexitarian). These factors presents further methodological challenges, limit comparability across studies and may contributes to variability in findings.

Additionally, the majority of available studies focus predominantly on short-term maternal and neonatal outcomes, with limited data on long-term effects on offspring health, growth, and development. Confounding variables such as socioeconomic status, education level, supplement use, and access to healthcare are also frequently underreported or uncontrolled, thereby introducing potential bias. Moreover, residual confounding and healthy-user bias may lead to overestimation of protective associations, while exposure misclassification due to self-reported dietary assessment may attenuate true relationships toward the null. Reverse causality is also possible, as women with higher cardiometabolic risk may modify their diet during pregnancy. Taken together, these limitations suggest that current conclusions should be interpreted cautiously, as observed associations may reflect correlated lifestyle or dietary quality factors rather than direct effects of plant-based diets per se. These limitations underscore the complexity of interpreting current evidence and highlight the need for high-quality prospective studies and randomized controlled trials with standardized dietary definitions and comprehensive adjustment for confounding variables to better clarify the causal role of PBDs in pregnancy-related outcomes in future research.

## Conclusion

When appropriately planned and supported with essential micronutrients such as vitamin B12, iron, calcium, and high-quality protein, PBDs may serve as a safe and health-promoting dietary approach during pregnancy. Owing to their high fiber content and low levels of saturated fat and refined sugars, PBDs have the potential to support favorable maternal metabolic health and fetal development. However, restrictive or poorly supplemented PBDs may increase the risk of micronutrient deficiencies, particularly in vulnerable populations. Importantly, current evidence on PBDs and pregnancy-related outcomes is derived predominantly from observational studies and does not allow firm causal conclusions. Existing data do not consistently demonstrate superior pregnancy outcomes among individuals adhering to PBDs compared to omnivorous diets, and the data specific to pregnant vegetarians and vegans remain limited. Therefore, while PBDs may be viable during pregnancy, conclusions should be interpreted with caution. Further high-quality randomized controlled trials and longitudinal studies with standardized definitions of plant-based dietary patterns are needed to clarify their safety, efficacy, and long-term effects on maternal and fetal health.

## Key References

- Zhu Y, Zheng Q, Huang L, Jiang X, Gao X, Li J, et al. The effects of plant-based dietary patterns on the risk of developing gestational diabetes mellitus: A systematic review and meta-analysis. Plos one. 2023;18(10):e0291732.10.1371/journal.pone.0291732
  - This systematic review and meta-analysis provides the most relevant evidence on PBDs and GDM, showing that adherence to healthy PBDs significantly lowers GDM risk, whereas unhealthy PBDs have a weaker effect, underscoring the importance of diet quality in pregnancy. These protective effects are likely mediated through improved insulin sensitivity, reduced inflammation, and beneficial modulation of gut microbiota.
- Mitsunami M, Wang S, Soria-Contreras DC, Mínguez-Alarcón L, Ortiz-Panozo E, Stuart JJ, et al. Prepregnancy plant-based diets and risk of hypertensive disorders of pregnancy. American Journal of Obstetrics and Gynecology. 2023.10.1016/j.ajog.2023.07.057
  - This large prospective cohort from the Nurses’ Health Study II provides high-quality evidence that greater preconception adherence to healthy PBDs lowers the risk of HDP, particularly gestational hypertension, with much of the benefit mediated by better weight control.
- Li T, He Y, Wang N, Feng C, Zhou P, Qi Y, et al. Maternal dietary patterns during pregnancy and birth weight: a prospective cohort study. Nutrition Journal. 2024;23(1):100. 10.1186/s12937-024-01001-8
  - This large cohort offers a current approach, indicating that PBDs are not associated with low birth weight, although carbohydrate-rich patterns were related to macrosomia. Importantly, this study highlights that well-planned plant-based diets do not adversely affect birth weight.

## Data Availability

No datasets were generated or analysed during the current study.
